# Infants’ neural responses to facial emotion in the prefrontal cortex are correlated with temperament: a functional near-infrared spectroscopy study

**DOI:** 10.3389/fpsyg.2015.00922

**Published:** 2015-07-20

**Authors:** Miranda M. Ravicz, Katherine L. Perdue, Alissa Westerlund, Ross E. Vanderwert, Charles A. Nelson

**Affiliations:** ^1^Laboratories of Cognitive Neuroscience, Division of Developmental Medicine, Boston Children’s Hospital, BostonMA, USA; ^2^Department of Pediatrics, Harvard Medical School, BostonMA, USA; ^3^Harvard Graduate School of Education, CambridgeMA, USA

**Keywords:** functional near-infrared spectroscopy, infancy, temperament, negative emotionality, emotion, face processing, prefrontal cortex

## Abstract

Accurate decoding of facial expressions is critical for human communication, particularly during infancy, before formal language has developed. Different facial emotions elicit distinct neural responses within the first months of life. However, there are broad individual differences in such responses, so that the same emotional expression can elicit different brain responses in different infants. In this study, we sought to investigate such differences in the processing of emotional faces by analyzing infants’s cortical metabolic responses to face stimuli and examining whether individual differences in these responses might vary as a function of infant temperament. Seven-month-old infants (*N* = 24) were shown photographs of women portraying happy expressions, and neural activity was recorded using functional near-infrared spectroscopy (fNIRS). Temperament data were collected using the Revised Infant Behavior Questionnaire Short Form, which assesses the broad temperament factors of Surgency/Extraversion (S/E), Negative Emotionality (NE), and Orienting/Regulation (O/R). We observed that oxyhemoglobin (oxyHb) responses to happy face stimuli were negatively correlated with infant temperament factors in channels over the left prefrontal cortex (uncorrected for multiple comparisons). To investigate the brain activity underlying this association, and to explore the use of fNIRS in measuring cortical asymmetry, we analyzed hemispheric asymmetry with respect to temperament groups. Results showed preferential activation of the left hemisphere in low-NE infants in response to smiling faces. These results suggest that individual differences in temperament are associated with differential prefrontal oxyHb responses to faces. Overall, these analyses contribute to our current understanding of face processing during infancy, demonstrate the use of fNIRS in measuring prefrontal asymmetry, and illuminate the neural correlates of face processing as modulated by temperament.

## Introduction

Much of human communication is unspoken. When we are angry or fearful or happy, those emotions are often reflected in our faces, and we observe others’ facial expressions in order to gather information about our social environment (Adolphs, 2002; Blair, 2003). Emotion processing is studied in infants in order to better understand where, when, and how this specialized ability develops. At the level of individuals, there are differences in the strength and sensitivity of neural responses to emotional faces (Etkin et al., 2004; Jessen and Grossmann, 2015). The present study examines the relation between infants’ temperaments and their neural responses to happy face stimuli.

Research on face processing in infants has frequently focused on responses to fearful faces, and there are several compelling reasons why that is the case. The fear circuit is one of the clearest and best-documented brain circuits, and it develops at an early age in humans (LeDoux, 2000; Leppänen and Nelson, 2012). Between 5 and 7 months, infants develop a proclivity to respond preferentially to fearful faces; for example, 7-month-old infants spend more time scanning fearful faces than neutral or happy faces, and they show greater brain activity in certain face processing areas when looking at fearful faces (Nelson and Dolgin, 1985; Leppänen et al., 2007; Hoehl et al., 2008; Vanderwert and Nelson, 2014). As early as 7 months of age, infants’ brain responses to fearful faces versus happy faces are distinct, with greater attention allocated to fearful faces, even when the infants do not consciously perceive the faces (Jessen and Grossmann, 2015). Research suggests that further developmental changes in emotional face processing occur between 7 and 12 months. ERP analysis shows that 7-month-old infants allocate greater attention to happy faces versus angry faces, whereas 12-month-old infants show the opposite preference (Grossmann et al., 2007). Further study is required to understand how the neural architecture underlying emotional face processing develops over the first year of life.

In the present study, we analyze infants’ neural responses to happy faces. A happy face is likely the first expression that an infant sees in the world, and the facial expression most commonly experienced from a very early age. Despite this relevance to early life experience, little is known about the development of the neural architecture involved in processing happy faces.

**FIGURE 1:**
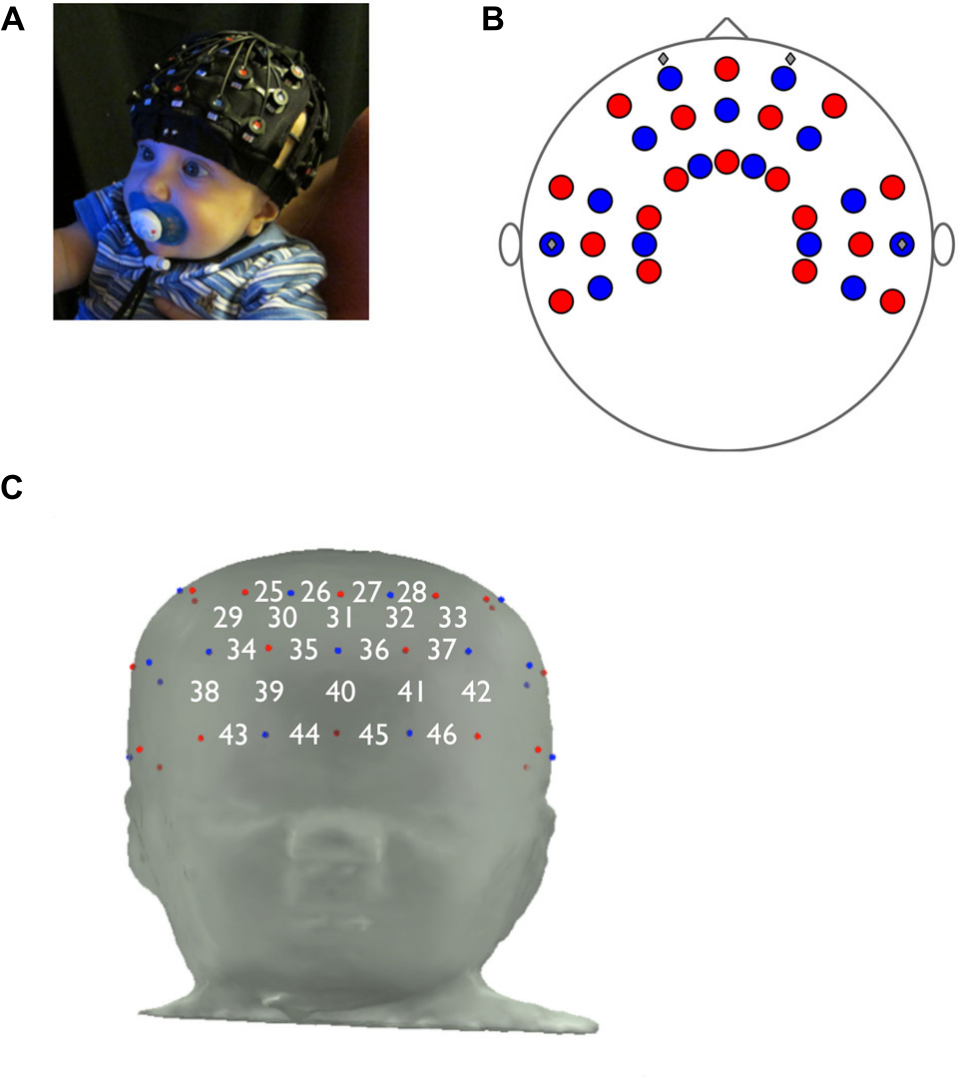
**Functional near-infrared spectroscopy probe design. (A)** Proper placement of fNIRS cap on infant. **(B)** fNIRS probe, top view. **(C)** Prefrontal panel of fNIRS probe, consisting of 22 channels (emitter-detector pairs), and the approximate locations of channels on the infant forehead.

Over the past several decades, researchers have developed a number of tools to study the developing brain and draw inferences about the neural bases of perceptual and cognitive functions. An emergent technique, fNIRS, is a non-invasive and infant-friendly methodology that measures changes in hemoglobin concentrations as an indicator of localized brain activity. As with fMRI, the fNIRS methodology assumes that increased oxyHb concentration and decreased deoxyHb concentration correspond to increased local brain activity (Lloyd-Fox et al., 2010). The fNIRS hardware is relatively inexpensive and portable, and the optodes (i.e., emitters and detectors) are arranged in a wearable cap, which is much more tolerable for infants and relatively more robust to movement artifact than is the fMRI scanner. Moreover, because fNIRS data can be collected in awake, behaving infants, this method allows for more ecologically valid experimental tasks (**Figure 1A**). fNIRS has better spatial resolution than EEG techniques, and better temporal resolution than fMRI. It is important to note, however, that because the brain topography is less well mapped for infants than for adults, it is difficult to know which brain regions underlie specific channels in the fNIRS probe, although recent modeling work has begun to address this issue (Lloyd-Fox et al., 2014). One drawback to fNIRS, in comparison with fMRI, is that it is limited to interrogating the cortical surface. However, as fNIRS methodology continues to evolve, increasingly complex experimental paradigms will provide critical insights into the functions of the developing brain.

Recently, fNIRS studies have examined the hemodynamic response to face stimuli, confirming that a distinct brain response to faces can be measured with this methodology (Blasi et al., 2007; Lloyd-Fox et al., 2009; Vanderwert and Nelson, 2014). Nakato et al. (2011) recorded fNIRS responses over the temporal regions, demonstrating that infant hemodynamic responses to distinct types of facial stimuli (happy and angry) are significantly different. They found that the left temporal area overlying the STS was significantly activated when infants viewed happy faces, and the right temporal area was significantly activated for angry faces. These results suggest that emotions are processed differentially over regions associated with face processing in the temporal cortices; however, this study did not examine evaluative processing of emotional faces in the PFC.

Another infant fNIRS study provides evidence that the OFC is involved with face processing and emotional arousal, and that these brain functions are measurable with fNIRS methodology by 12 months of age (Minagawa-Kawai et al., 2009). In this study, infants were shown videos of their mothers and of an unfamiliar woman, posing with neutral and smiling expressions. The results found that, in a channel in the medial PFC, there was a significant difference in oxyHb activation in the smiling condition versus the neutral condition when the infant watched the video of his/her own mother. The same effect was found for the smiling versus neutral unfamiliar woman stimuli, with marginal significance. These data indicate that there is a detectable response to smiling faces in the medial OFC.

More recently, an fNIRS study of 6- and 7-month-old infants at high and low risk for autism provides further insight into how infants process smiling versus neutral faces (Fox et al., 2013). The study examined oxy- and deoxyHb responses to videos of female faces changing from a neutral expression to a smiling one. In three channels over the right frontal cortex, there was a main effect of emotion, in which the oxyHb response to smiling faces was greater than the response to the neutral expression. In three channels over the left frontal cortex, infants showed greater (that is, more negative) deoxyHb responses to smiling versus neutral faces. Taken together, these studies demonstrate that fNIRS methodology can detect stimulus-driven responses to happy faces in distinct channels over the frontal cortex.

In the research conducted to date, infants’ data were pooled and examined at the group level, with little attention paid to individual differences. Thus, in the current study we sought to add an individual difference dimension — specifically, to examine whether differences in infants’ temperament might be associated with differences in infants’ hemodynamic response. Temperament refers to a biologically determined disposition toward certain behaviors or feelings. A person’s temperament, observable during infancy and relatively stable over the lifetime (Fox et al., 2001), affects susceptibility to certain emotional states, intensity of emotion, and the ability to regulate emotional responses. Evidence suggests that temperamental biases are determined by genes influencing neurochemistry and neuroanatomy, and by the prenatal environment (Kagan and Snidman, 2004).

Childhood environment and early experiences, as well as genetic expression that is modified throughout development, will determine how a child’s temperament manifests as personality traits, and whether those traits will change over time as the individual develops from infancy to childhood to adolescence and eventually into adulthood (Kandler et al., 2013). Infant temperament has been shown to be moderately predictive of temperament in toddlerhood and early childhood, with strong longitudinal correlations for the factor levels of S/E and Negative Affect (Fox et al., 2001; Putnam et al., 2008). Furthermore, a large longitudinal study showed that temperament groups at age 3 — specifically the dimensions of undercontrolled, inhibited, and well-adjusted — predict personality style at age 18, and that temperament at age 3 predicts the quality of interpersonal relationships and social support, as well as the incidence of unemployment, psychiatric disorders, and criminal behavior, at age 21 (Caspi, 2000). Though the effect size for each of these connections was only small to medium, Caspi (2000) argues that the association between temperament during toddlerhood and multiple independent measures of psychosocial functioning in young adulthood provides important evidence for the developmental continuity of temperament.

Previous investigations have found that infant temperament is associated with individual differences in emotional face processing, specifically in the amplitude and latency of the Nc, an ERP component associated with allocation of attention. Martinos et al. (2012) found that, from 3 to 13 months, infants with higher NE allocated greater attention (as indexed by the Nc) to happy faces than to fearful faces. This pattern of findings has not always been consistent, however; de Haan et al. (2004) found that more fearful infants showed a larger Nc in response to fearful faces than to happy faces. Martinos et al. (2012) attributed this discrepancy to methodological differences in temperament assessment or ERP measurement, or to the different age ranges of the subjects.

Temperament has been robustly associated with differences in EEG activity observed over the left and right frontal scalp (known as ‘frontal EEG asymmetry’). According to Davidson’s (1993) ‘motivational model’ of EEG asymmetry, relatively greater activity over the left (versus right) frontal lobe is associated with ‘approach’ orientation or behavior. Relatively greater right (versus left) frontal activation is associated with ‘withdrawal’ orientation or behavior. Infants who demonstrate withdrawal behavior are reticent and distressed when presented with unknown people or novel objects; infants demonstrating approach behavior readily approach new people and toys, and remain unperturbed in stressful situations. Studies have examined both state-dependent effects (for example, smiling can increase relative left frontal activation) and trait-dependent effects (Ekman and Davidson, 1993; Coan and Allen, 2003). The trait-dependent effects are relatively stable measures of individual temperament, with high internal consistency and acceptable test–retest stability (Tomarken et al., 1992; Fox et al., 2001). The measurement of frontal asymmetry can provide insight into the biological basis of temperament.

Many studies of frontal asymmetry have investigated the cortical response to emotional faces (Davidson and Fox, 1982; reviewed in Davidson, 1993). A meta-analysis of fMRI research analyzing adult brain responses to photos of emotional faces found that there were significant hemispheric interactions between the category of facial expression (approach- versus avoidance-inducing) and activity in the PFC (Fusar-Poli et al., 2009). Frontal asymmetry in response to emotional faces provides information about how the PFC is involved in emotion perception and how the response is modulated by individual temperament.

Preliminary work suggests that state- and trait-related asymmetry can also be studied using fNIRS. In a study by Tuscan et al. (2013), young adult subjects were asked to complete three tasks while fNIRS activity over the PFC was recorded during the different task manipulations: conversing with strangers, planning a 5-minute speech, and delivering this speech. Subjects reported their subjective anxiety levels after each of the three tasks, and each subject’s trait anxiety was evaluated using the Social Phobia and Anxiety Inventory (SPAI). During the anticipation and speech phases, subjects experienced relatively greater blood volume and oxyHb concentrations in the right relative to the left hemisphere. Further, the participants who were identified as higher in anxiety showed a trend toward greater right frontal activation relative to low-anxious subjects (Tuscan et al., 2013). This observed effect is analogous to right frontal EEG asymmetry, which is associated with withdrawal behavior and would be predicted in tasks designed to induce social stress.

We selected channels overlying the PFC as the region of interest. The PFC is involved in distinguishing facial emotions (Leppänen and Nelson, 2009) and in emotional regulation (for example, Tranel et al., 2002), roles that are likely modulated by reciprocal connections with subcortical structures involved in emotion perception, particularly the amygdala and superior colliculus (Leppänen and Nelson, 2009). We were not able to collect data from the OFA or FFA because these primary face processing areas lie too deep below the cortical surface to detect with fNIRS (Otsuka et al., 2007), and because the fNIRS probe used in this study lies over the infant’s temporal and prefrontal cortices. Some fNIRS studies of infant face processing have examined the area overlying the STS, another face processing area (Lloyd-Fox et al., 2009; Nakato et al., 2011). However, because we were interested in measuring frontal asymmetry in response to facial stimuli, we chose to focus on the PFC. Furthermore, Minagawa-Kawai et al. (2009) showed significant activity in a region of the PFC for infants’ response to smiling faces.

We hypothesized that there would be channels in the prefrontal panel in which hemoglobin activity would be correlated with temperament. Specifically, we hypothesized that the temperament factors of S/E and O/R would be positively correlated with brain activation, that NE would be negatively correlated with brain activation. Furthermore, to explore the capacity of fNIRS methodology to record relative asymmetry in oxyHb activity between left and right hemispheres, we analyzed the interactions of temperament and hemisphere. We hypothesized that infants with higher S/E scores would show relatively greater left frontal activity, and infants with higher NE scores would show relatively greater right frontal activity. These analyses contribute to our current understanding of face processing during infancy, investigate the use of fNIRS in measuring prefrontal asymmetry, and examine the neural correlates of face processing as modulated by temperament.

## Materials and Methods

### Participants

Twenty-four 7-month-old infants were included in the study (mean age 212 ± 1.0 days, range 205–221 days; 11 females). Twenty additional infants were tested but were excluded from the study for incorrect optode placement (*n* = 7), for more than 25% of channels in the prefrontal panel rejected for artifact (*n* = 6), for equipment failure (*n* = 5), or for movement artifact (*n* = 2). The 45% attrition rate is comparable to the rate in other infant fNIRS studies (Lloyd-Fox et al., 2010). Infants who were included or excluded from the study did not differ in measures of S/E, *t*(42) = 1.16, *p* = 0.252, NE, *t*(42) = -1.02, *p* = 0.314, or O/R, *t*(42) = 0.196, *p* = 0.846. Infants were recruited from a registry of local births set up by the Laboratories of Cognitive Neuroscience. Infants were excluded from recruitment if they were born more than 3 weeks before their due date, or if they had any neurological disorders, including neurological trauma, developmental delay, uncorrected vision difficulty, or birth-related complications. Written informed consent was obtained from each infant’s parent or primary caregiver prior to the start of the experiment, and the experimental protocol was approved by the Boston Children’s Hospital Institutional Review Board. Written informed consent was also obtained from the parent for use of the photo in **Figure 1A**.

### Infant Behavior Questionnaire

We used the R-IBQsf (Putnam et al., 2013), a parent-report measure of temperament that was completed by the mother or primary caregiver of each subject prior to the visit. The tool is validated for 3- to 12-month-old infants and has showed adequate internal consistency, inter-rater reliability between mothers and fathers, and convergence with laboratory observational assessments (Gartstein and Rothbart, 2003; Parade and Leerkes, 2008; Putnam et al., 2013).

The R-IBQsf consists of 14 subscales that load onto three broad temperament factors, as derived from principle factor analysis (Gartstein and Rothbart, 2003). The S/E factor includes temperament subscales of activity level, vocal reactivity, smiling and laughing, high intensity pleasure, perceptual sensitivity, and approach. The NE factor includes questions about fear, distress to limitations, sadness, and (loading negatively) falling reactivity. The third factor, O/R, includes the subscales of soothability, duration of orienting, cuddliness, and low intensity pleasure. Gartstein and Rothbart (2003) found low bivariate correlations between the factors.

### NIRS Recording

Hemodynamic responses were recorded using a multichannel optical topography NIRS instrument (ETG-4000, Hitachi Medical Corporation, Tokyo, Japan). Near-infrared light at 695 and 830 nm was conveyed to the emitting optodes via optical fibers and shined onto the scalp. The light that passed back through the scalp was then conveyed from the detecting optodes via optical fibers to photodetectors that measured the intensity of the attenuated light. The inter-optode distance was fixed at 3.0 cm. Data were sampled every 100 ms (10 Hz). The fNIRS probe was mounted inside a flexible cap, which was placed on the infant’s head and worn for the duration of the experiment (**Figure 1A**). The probe was customized for this experiment and included 46 channels, each consisting of an emitter and detector combination, that were positioned over the frontal, temporal, and parietal cortices (**Figure 1B**). In this study the area of interest was the PFC, underlying channels 25 through 46 (**Figure 1C**).

To ensure precise and consistent spatial resolution in the fNIRS data, we adhered to stringent criteria for hat placement. During each session, photos were taken of the cap placement (frontal and lateral views), and the photos were reviewed by multiple experimenters. Subjects were excluded for incorrect hat placement if the cap was shifted by more than 1 cm in any direction (left, right, up, or down).

### Task and Stimuli

Infants completed the task while sitting on their parent’s lap. Parents were asked not to speak to the infant during the experiment, and they wore a visor to prevent any parental response to the visual stimuli from influencing the infant’s reaction. The infant was seated approximately 60 cm from a 17-inch computer monitor. The stimuli were 16.5 cm high (visual angle: 14.3°) and 14 cm wide (visual angle: 12.2°). The testing room was soundproof and the lights were dimmed during the experiment to a standardized brightness. An experimenter sat next to the infant and parent and redirected the infant’s attention to the screen before the start of each trial. In order to minimize data attrition, parents were asked to select a time for the visit when the infant was typically alert and content, and the infant was allowed to take breaks during the session as needed.

**FIGURE 2:**
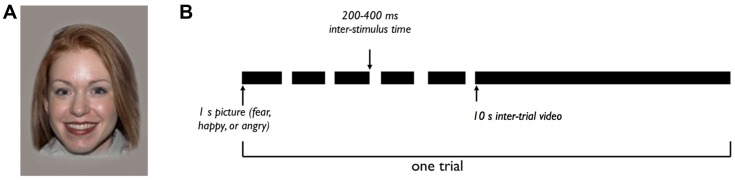
**Experimental design. (A)** Example ‘happy’ stimulus. Each trial consists of five images like this one, with different female models portraying a happy expression. **(B)** Each trial includes five images, each presented for 1 s with a 200–400 ms inter-stimulus time. The five images in a trial represent a single emotional category (fearful, happy, or angry), with five different female models portraying the emotional expression. After the fifth image, a 10 s inter-trial video is shown, for a total trial time of about 16 s. The experiment consists of 30 total trials, with 10 trials of each emotional category.

The stimuli were images of female models displaying happy, fearful, and angry facial expressions (**Figure 2A**), selected from the NimStim Face Stimulus Set (Tottenham et al., 2009). The stimuli were presented using the E-Prime Application Suite for Psychology (E-Prime 2.0, Psychology Software Tools, Sharpsburg, PA, USA).

The experiment consisted of a maximum of 30 trials, each of which included five images. The five images in a single trial were of the same emotional category (happy, fearful, or angry), portrayed by different models. Each image was shown for 1 s, with a randomly generated 200–400 ms inter-stimulus time. After the set of five images, a video of non-face shapes was shown for 10 s, resulting in a total trial length of 16 s (**Figure 2B**). There were 10 trials of each of the three emotional categories, for a total of 30 trials. The session ended when a participant viewed all 30 trials, or if the participant grew restless or upset. The order of stimulus presentation was counterbalanced across subjects.

### Data Processing

Oxy- and deoxyHb data from ‘happy’ trials were included in this analysis. Subjects viewed a maximum number of 10 ‘happy’ trials; they viewed fewer than 10 trials if they refused to complete the entire task, or if they looked away from the stimulus during a given trial. For each subject, a video recording of the experimental proceedings was coded oﬄine using SuperCoder software (SuperCoder 1.7.1, Purdue University, West Lafayette, IN, USA) by observers who were blind to the emotional category. Inter-rater reliability was maintained at 0.90 with 15% coding overlap. Trials in which the infant was not looking at the stimulus for at least 50% of the time the stimulus was on the screen were excluded. Trials were not excluded for failure to look during the inter-trial video. Infants completed an average of 8 ± 0.3 ‘happy’ trials (*N* = 24), and the range of valid trials was 5–10. We used an *a priori* threshold of three ‘happy’ trials for inclusion in the final sample, based on a previous study of face processing in infants (Lloyd-Fox et al., 2013).

Functional near-infrared spectroscopy data were processed using HOMER2 (MGH-Martinos Center for Biomedical Imaging, Boston, MA, USA), a MATLAB (The MathWorks, Inc., Natick, MA, USA) software package. The attenuated light intensities measured by the detecting optode at each channel were converted to optical density units, and then filtered using a band pass filter with a passband from 0.050–0.80 Hz. They were also processed using wavelet motion correction as implemented in HOMER2 with an interquartile range of 0.5 (Cooper et al., 2012; Molavi and Dumont, 2012). The filtered data were used to calculate the change in concentration of each hemoglobin chromophore according to the modified Beer–Lambert Law (Delpy et al., 1988), assuming a pathlength factor of 5 (Duncan et al., 1995). Chromophore concentrations were baseline corrected using the 2 s prior to stimulus presentation, as in previous fNIRS studies (for example, Watanabe et al., 2008).

In the data processing stream, channels in the fNIRS probe were excluded for artifact if the magnitude of the signal was greater than 98% or less than 2% of the total range for longer than 5 s during the recording. Subjects with more than 25% of channels in the region of interest marked unusable were excluded from further analysis. For this experimental probe design, there were 22 channels in the prefrontal panel, and subjects with more than five channels marked unusable were excluded.

### Statistical Analyses

Statistical tests were conducted using IBM SPSS Statistics 21.0 (IBM Corporation, Armonk, NY, USA). One-sample *t*-tests were conducted to determine if the maximum changes in oxyHb or deoxyHb concentration in the channels of interest were significantly different from baseline levels. A time window of interest was selected between 0 and 10 s, with *t* = 0 s corresponding to the time of stimulus onset. Baseline values were measured between -2 and 0 s, as in previous fNIRS studies (for example, Watanabe et al., 2008).

Bivariate Pearson correlations were conducted between the three temperament factors (S/E, NE, and O/R). Pearson correlations were then calculated for channels in the region of interest to test for a relation between temperament and oxyHb and deoxyHb activity (maximum amplitude). Due to a significant correlation between S/E and O/R (see below), partial correlations were also conducted to test the independent relations between temperament and Hb activity. In order to fully explore the connection between happy faces and temperament, only the responses to happy face stimuli were included in these analyses.

Repeated measures analysis of variance was used to test for a hemispheric effect of temperament on oxyHb activity, with hemisphere (left versus right) as the within-subjects factor and the temperament group (low versus high) as the between-subjects factor. Activity in the left and right hemispheres was calculated as the mean value of the maximum change in oxyHb amplitude for two channels in the left hemisphere (36 and 41) and for two channels in the right hemisphere (35 and 39). A median split was used to divide subjects into low and high temperament groups for each factor (S/E, NE), as in previous studies (for example, Baehr et al., 1998; Hagemann et al., 1999; Hagemann, 2004).

## Results

### Prefrontal Activation in Response to Happy Face Stimuli

**FIGURE 3:**
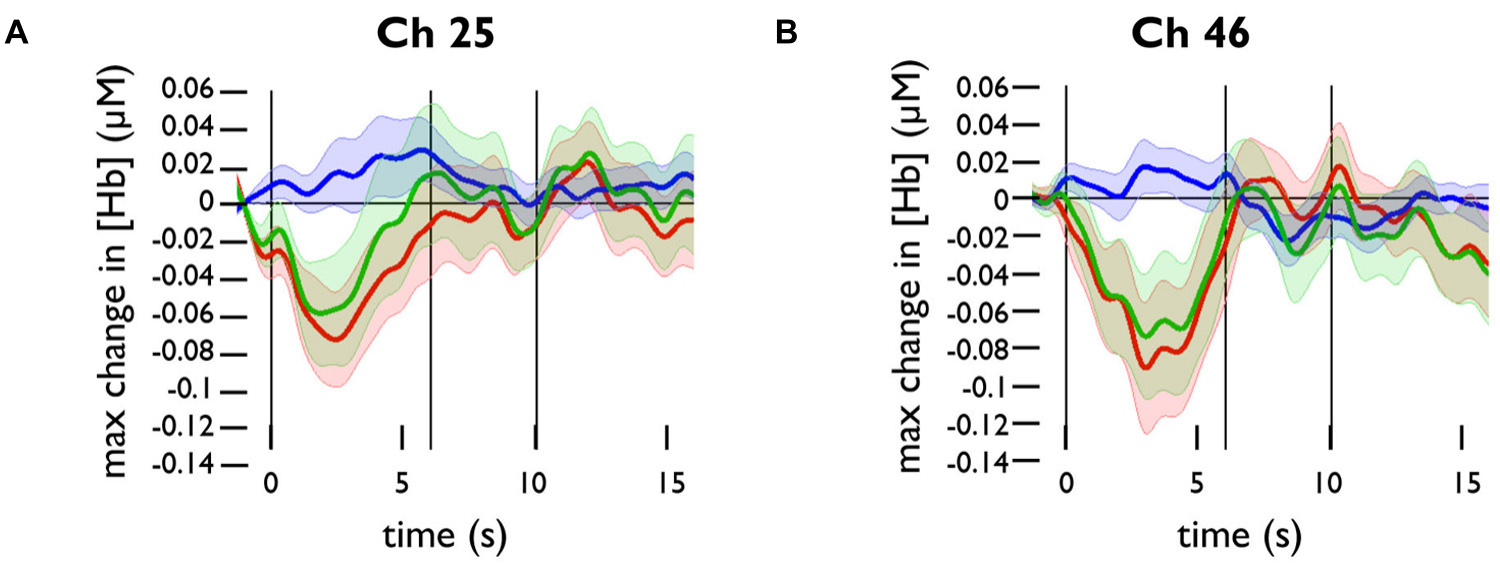
**Functional near-infrared spectroscopy activation in response to happy face stimuli in channels 25 and 46.** Grand averaged time courses of the changes in concentration of oxy- (red), deoxy- (blue), and totalHb (green) in **(A)** channel 25 and **(B)** channel 46.

We plotted the grand averaged time courses of the changes in concentration of oxy-, deoxy-, and totalHb for the channels in the prefrontal panel. The responses for channels 25 and 46 are shown in **Figure 3**. Based on these responses, we selected a time range of 0–10 s for analysis.

To test whether channels overlying the PFC were significantly activated by happy face stimuli, we conducted one-sample *t*-tests for the maximum change in oxyHb and deoxyHb concentrations in these channels. The changes in Hb concentration were calculated relative to the baseline value at *t* = -2 to 0 s. Across subjects, there was a significant decrease in oxyHb concentration in channel 25, *t*(20) = -2.767, *p* = 0.012, and in channel 46, *t*(23) = -2.387, *p* = 0.026. There was a significant increase in deoxyHb concentration in channel 34, *t*(23) = 2.959, *p* = 0.007.

### Differential Brain Responses According to Temperament

In our sample, we found no significant correlation between NE and S/E or O/R, which is consistent with previous findings (Gartstein and Rothbart, 2003). We did find a significant correlation between S/E and O/R, *r*(22) = 0.674, *p* < 0.001.

We conducted Pearson correlations to test whether oxyHb and deoxyHb activity (calculated as maximum change in concentration from baseline) were correlated with temperament in the prefrontal channels. The results are shown in **Table 1**. A total of *N* = 24 subjects were tested, but because some channels did not have reliable data from all subjects, the number of subjects (*n*) tested for each channel is shown in the table. The temperament factor S/E was negatively correlated with oxyHb activity in channel 26, *r*(21) = -0.521, *p* = 0.011, and channel 32, *r*(21) = -0.457, *p* = 0.028; in these channels, infants with lower S/E scores showed greater activation in response to happy faces. Similarly, NE was negatively correlated with oxyHb in channel 36, *r*(22) = -0.414, *p* = 0.044, channel 41, *r*(22) = -0.476, *p* = 0.019, and channel 42, *r*(21) = -0.423, *p* = 0.044. O/R was negatively correlated with oxyHb activity in channel 27, *r*(19) = -0.525, *p* = 0.015, channel 28, *r*(19) = -0.685, *p* = 0.001, channel 32, *r*(21) = -0.423, *p* = 0.044, and channel 33, *r*(21) = -0.585, *p* = 0.003. Infants with lower NE scores and lower O/R scores, respectively, showed greater activation in response to happy faces than did infants with higher NE and O/R scores. In one channel (32), oxyHb activity was correlated with both S/E and O/R. DeoxyHb activity was correlated with S/E in channel 27, *r*(19) = -0.500, *p* = 0.021; with NE in channel 43, *r*(22) = 0.411, *p* = 0.046, and channel 45, *r*(22) = -0.430, *p* = 0.036; and with O/R in channel 28, *r*(19) = -0.525, *p* = 0.014.

**Table 1:** Summary of channels in which maximum change in concentrations of oxyHb or deoxyHb are significantly correlated with the temperament factors.

		oxyHb			deoxyHb		
Channel	*n*	S/E	NE	O/R	S/E	NE	O/R
26	23	-0.521^∗^					
27	21			-0.525^∗^	-0.500^∗^		
28	21			-0.685^∗∗^			-0.525^∗^
32	23	-0.457^∗^		-0.423^∗^			
33	23			-0.585^∗∗^			
36	24		-0.414^∗^				
41	24		-0.476^∗^				
42	23		-0.423^∗^				
43	24					0.411^∗^	
45	24					0.430^∗^	

*Values are Pearson correlations. S/E, Surgency/Extraversion; NE, Negative Emotionality; O/R, Orienting/Regulation. ^∗^*p* < 0.05; ^∗∗^*p* < 0.01.*

**FIGURE 4:**
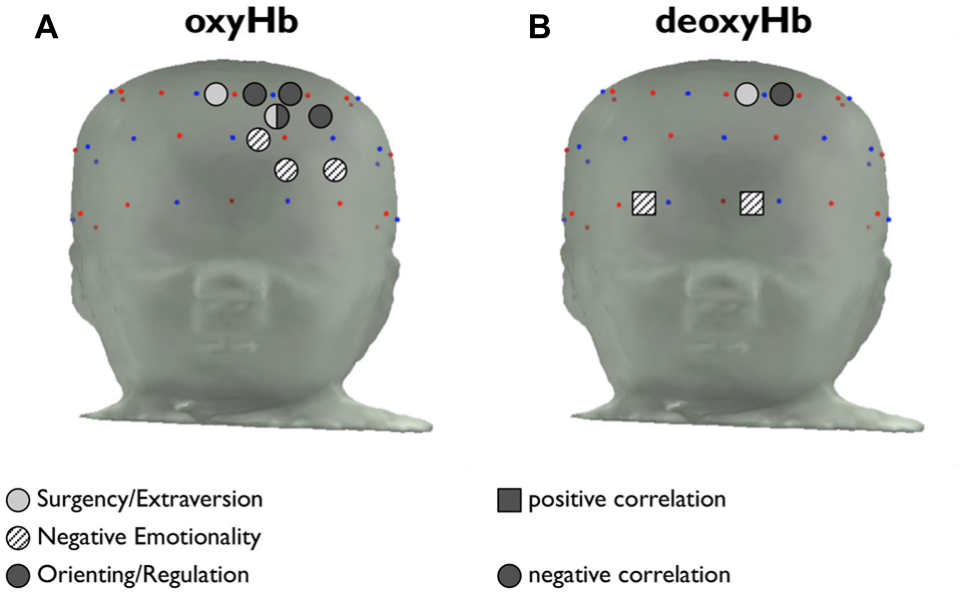
**Placement of channels in which temperament is correlated with hemoglobin activity. (A)** Channels in which the maximum change in amplitude from baseline of [oxyHb] is significantly correlated with S/E, NE, and O/R are indicated in light gray, patterned, and dark gray, respectively. Positive correlations are indicated with a square marker, and negative correlations are indicated with a circular marker. **(B)** Channels in which maximum change in amplitude from baseline of [deoxyHb] is significantly correlated with temperament factors.

The approximate locations of the correlated channels are shown in **Figure 4**. None of the correlations between temperament and concentration was significant at the level required to correct for multiple comparisons (*p* < 0.0008). However, the correlated channels are clustered by temperament group, suggesting a consistent pattern of activation. The four channels in which oxyHb activity is correlated with O/R are adjacent to one another, as are the three channels in which oxyHb is correlated with NE, as shown in **Figure 4A**. The two channels with significant correlations between S/E and oxyHb are nearly adjacent.

The channels in which deoxyHb concentration is correlated with temperament are shown in **Figure 4B**. The channels where deoxyHb activity is correlated with S/E and O/R overlap with regions where oxyHb activity is correlated with temperament, as would be expected. The channels in which NE is positively correlated with deoxyHb activity are located along the brow.

The three higher-order temperament factors (S/E, NE, and O/R) are, in theory, orthogonal and uncorrelated (Gartstein and Rothbart, 2003). However, because S/E and O/R were correlated for our data, we conducted partial correlations to examine the independent contributions of each factor. We found that, when controlling for S/E, oxyHb was significantly correlated with O/R in channel 28, *r*(18) = -0.607, *p* = 0.005, and channel 33, *r*(20) = -0.532, *p* = 0.011. The correlation approached significance in channel 44, *r*(21) = -0.403, *p* = 0.056. DeoxyHb was significantly correlated with O/R in channel 28, *r*(18) = -0.506, *p* = 0.023. When controlling for O/R, neither oxyHb nor deoxyHb was significantly correlated with S/E.

### Frontal Asymmetry Measured by fNIRS

We hypothesized that fNIRS imaging could detect an asymmetry effect in the prefrontal response to happy face stimuli. Individuals with a greater proclivity for ‘approach’ behaviors show relatively greater left frontal activation, while individuals who tend to display ‘withdrawal’ behaviors show relatively greater right frontal activation. Based on these established findings in both infants and adults (Davidson and Fox, 1982; Davidson, 1993; Fox et al., 2001; Coan and Allen, 2003), we hypothesized that subjects with higher S/E temperament scores would show greater relative left frontal activation. Subjects with higher NE scores would show greater relative right frontal activation.

#### Negative Emotionality

We first tested for a hemispheric effect between the high and low NE groups. We conducted an RM-ANOVA with the two hemispheres (left and right) as the within-subjects factor, and the temperament group (high or low NE) as the between-subjects factor. To calculate the oxyHb activation in each hemisphere, we averaged the activity in two channels on the left side (36 and 41) and two channels on the right side (35 and 39) of the prefrontal panel of the fNIRS probe. These channels were selected because they were located in symmetrical positions on the medial region of the prefrontal probe, an area commonly analyzed for hemispheric comparisons using fNIRS and EEG (Tuscan et al., 2013). Activity in channels 36 and 41 was significantly correlated with NE, so we expected to see an effect of temperament in this analysis.

There was a significant main effect of NE group, *F*(1,22) = 4.80, *p* = 0.039. The low-NE group had greater overall activation to happy faces (*M* = 0.070, SD = 0.137) compared to the high-NE group (*M* = -0.061, SD = 0.154) in the four channels of interest. This suggests that infants with less negative temperament are more responsive to images of happy faces than are infants with more negative temperament. There was no significant main effect of hemisphere, *F*(1,22) = 1.11, *p* > 0.05; the overall activation was not significantly different between the left hemisphere and the right hemisphere.

**FIGURE 5:**
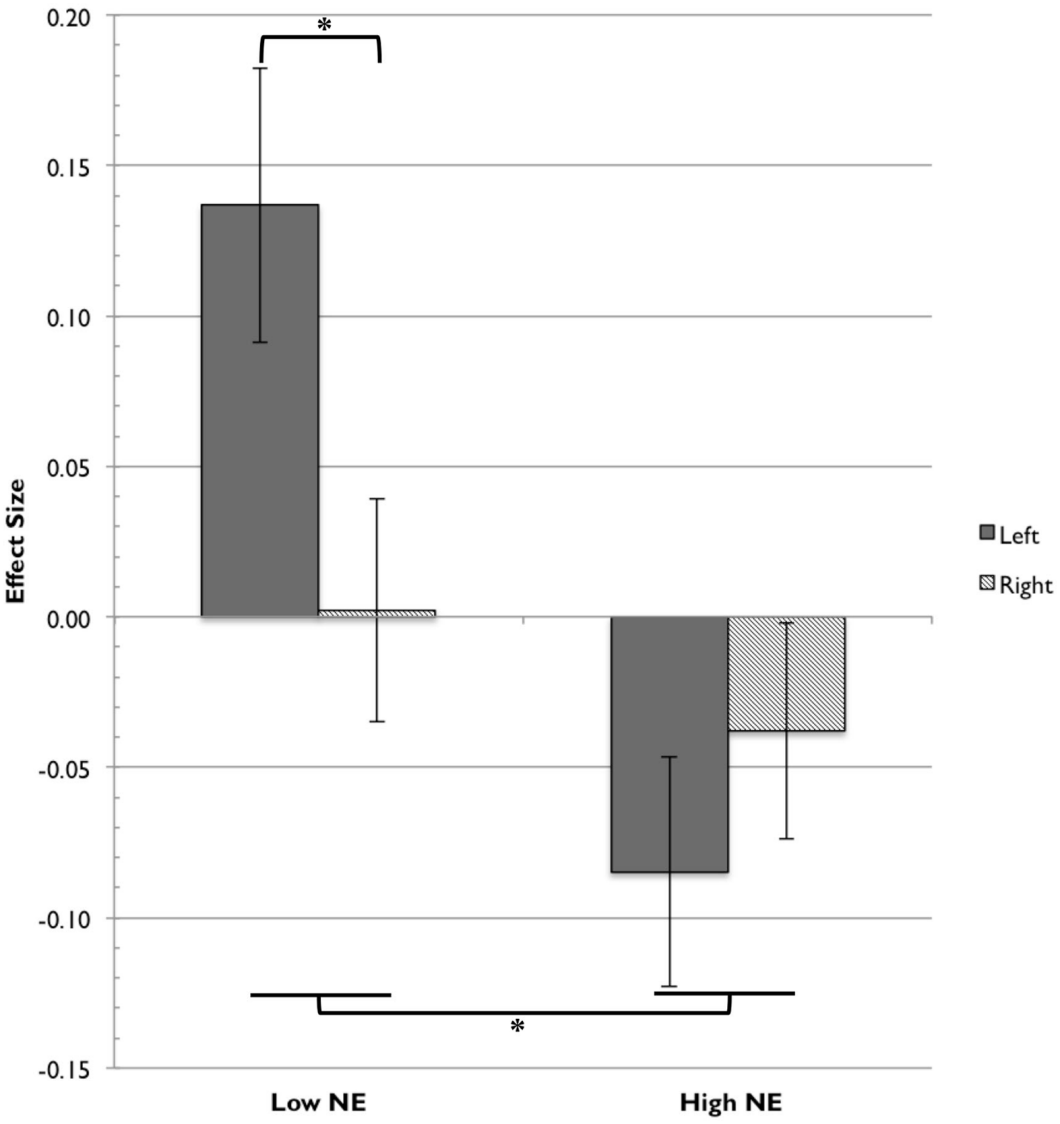
**Frontal fNIRS asymmetry in low NE and high NE groups.** For the low NE group, the left hemisphere (channels 36, 41) showed significantly greater activation relative to the right hemisphere (channels 35, 39). The low NE group showed significantly greater activation overall than the high NE group. Asterisks indicate significance at the level of *p* < 0.05.

The main effect of group was modified by a Group × Hemisphere interaction, *F*(1,22) = 4.75, *p* = 0.040. *A priori* pairwise comparisons testing hemispheric differences between the two temperament groups found that the low NE group and high NE group did not differ in right hemisphere activation, *t*(22) = 0.487, *p* = 0.631, but the two groups did differ significantly in left hemisphere activation, *t*(22) = 3.614, *p* = 0.002. Low-NE infants showed preferential activation in response to happy faces in the left hemisphere, while high-NE infants showed less overall activation in both hemispheres. The asymmetry effect is shown in **Figure 5**.

#### Surgency/Extraversion

We also conducted an RM-ANOVA with S/E group as the between-subjects factor. There were no significant main effects of S/E group, *F*(1,22) = 0.354, *p* = 0.56, or of hemisphere, *F*(1,22) = 1.03, *p* = 0.32, and no significant Group × Hemisphere interaction, *F*(1,22) = 2.69, *p* = 0.12.

## Discussion

### Key Findings

The objectives of the present study were to characterize the prefrontal hemodynamic response of 7-month-old infants to happy face stimuli, to analyze the relation between infants’ temperament and their brain responses to happy face stimuli, and to examine the capabilities of fNIRS methodology to provide information about frontal asymmetry. We showed that happy face stimuli elicited significant changes relative to baseline in oxyHb and deoxyHb concentrations in three channels (25, 34, and 46). Based on the time course of these responses, we selected a time range of interest of *t* = 0–10 s, with *t* = 0 s corresponding to the start of stimulus presentation. Through correlational analyses, we showed that the maximum change in both oxyHb and deoxyHb concentrations in response to happy faces was significantly correlated with S/E, NE, and O/R temperaments in channels overlying the left PFC. However, when controlling for the O/R factor, S/E is not correlated with oxy- or deoxyHb activity in any of the channels.

Further, we demonstrated that fNIRS can be used to study frontal asymmetry, a direction of analysis that is well-established in EEG literature but relatively unexplored in NIRS. We showed that there was a main effect of NE temperament group (low and high) modified by a Group × Hemisphere interaction. The low-NE infants preferentially activated the left hemisphere in response to happy faces. High-NE infants did not show this lateralization effect, and the overall activation for low-NE infants was higher than for high-NE infants. We had hypothesized that high-S/E infants would show relatively greater left frontal activation, but there were no notable effects of S/E group and hemisphere.

### Hemodynamic Differences from Baseline

The typical NIRS response in adults shows an increase in oxyHb, and a corresponding decrease in deoxyHb that is relatively smaller in magnitude. This response at a given channel is thought to indicate an increase in brain activation in the cortical region underlying the channel. The activation in channels 25, 34, and 46 did not follow this pattern of typical activation; channels 25 and 46 showed a significant decrease in oxyHb concentration, and channel 34 showed a significant increase in deoxyHb concentration. Atypical hemodynamic patterns in infant brains (such as simultaneous increases in all three chromophores) have been described previously, but they are believed to be the result of immature neurovascular coupling (Lloyd-Fox et al., 2010). It has also been proposed that a decrease in oxyHb and corresponding increase in deoxyHb would indicate local decreased neural activity as compared to baseline, just as a decrease in fMRI BOLD signal is considered to represent brain deactivation (Fransson et al., 1999; Sakatani et al., 2006), or that this inverse pattern indicates activation in an adjacent brain region (“focal activation/surround deactivation”; Pfurtscheller et al., 2010).

Because channels 25, 34, and 46 are not adjacent to one another, it is difficult to draw conclusions about the implications of these activations. It is noteworthy, however, that channels 25 and 46 are located on the edges of the region in which oxyHb activity is correlated with temperament factors (channels 26, 27, 28, 32, 33, 36, 41, and 42). This suggests that the broad region could be involved with emotional face processing, with some areas activated across all subjects and other areas differentially activated as a result of individual differences — in this case, infant temperament.

It is unclear whether the observed responses were specific to happy face stimuli. Future research will compare the hemodynamic responses to happy face stimuli with the responses to other emotional expressions (fearful, angry, and neutral).

Previous studies in fNIRS have found a significant neural response to facial stimuli in the medial prefrontal region. In one fNIRS study that analyzed the oxyHb response to happy face stimuli, there was significant activation relative to neutral stimuli in a single channel overlying the medial PFC, demonstrating that there is a measurable response to happy face stimuli in this region (Minagawa-Kawai et al., 2009). Similarly, a second study showed a greater response to smiling faces than to neutral faces in six frontal channels, with three channels over the right frontal cortex showing a greater oxyHb response to happy faces, and three channels over the left frontal cortex showing a greater (more negative) deoxyHb response to happy faces (Fox et al., 2013). Based on these previous findings, we were surprised that we did not observe greater prefrontal activation to happy face stimuli. However, because we chose to analyze the response to facial stimuli relative to a non-face baseline, rather than to a neutral face baseline, the results are not directly comparable.

### Temperament Correlated with OxyHb and DeoxyHb Activity

As hypothesized, temperament was correlated with hemodynamic activity in regions of the PFC. We chose not to correct these correlational results for multiple comparisons, given the exploratory nature of this analysis. However, the finding that local hemoglobin activation in these channels correlates with temperament is particularly compelling due to the clustering of channels by temperament group. The correlated activity is found in contiguous regions, rather than in scattered or isolated channels.

We would expect that activation to happy faces would be negatively correlated with NE. This study shows that, in these channels, infants with greater negative affect show less activation to happy face stimuli, while infants with less negative affect show greater neural activation. These results suggest that low-NE infants tend to be more responsive to happy faces.

It is more surprising that S/E was also negatively correlated with the oxyHb response to happy faces. We hypothesized that infants with higher S/E scores — infants who are more approach oriented — would show greater activation to happy faces. When we assumed that the three temperament factors were independent (Gartstein and Rothbart, 2003), this hypothesis was unconfirmed. However, when we controlled for O/R, there were no channels where S/E was correlated with oxyHb or deoxyHb activity. This suggests that the observed negative correlation between S/E and temperament was primarily driven by the O/R factor.

We noted that the channels in which NE was correlated with oxyHb activity were medial relative to the channels in which O/R was correlated with oxyHb activity. Previous work using fNIRS, fMRI, and other brain imaging technologies has shown a functional division in the infant PFC between the mPFC and lPFC. The mPFC has reciprocal connections with the amygdala, hippocampus, and temporal cortex — regions implicated in emotion, memory, and sensory processing — while the lPFC has reciprocal connections with motor regions, the cingulate cortex, and the parietal cortex. Broadly, the mPFC is involved with emotional processes, and the lPFC is involved with cognitive processes (Grossmann, 2013). The present findings are consistent with this distinction. The temperament factor of NE measures infants’ emotional proclivities (including fear and sadness), and the channels where NE is correlated with brain activity are relatively medial. The O/R factor measures attentional and regulatory tendencies, which would be consistent with cognitive processing in the left lateral PFC, and the channels where O/R is correlated with oxyHb activity are relatively lateral.

All of the channels except one (channel 26, correlated with S/E) are located in the left hemisphere. As described in the introduction, activation of the left hemisphere has been associated with motivation to approach (versus withdraw). We would expect that non-threatening face stimuli, such as the images used in this study, would elicit an ‘approach’ response in some individuals. It makes sense, then, that the subjects’ temperamental biases in their responses to happy face stimuli appeared to be driven by activation in the left hemisphere.

The correlations between temperament factors and deoxyHb activity are difficult to interpret. Most fNIRS studies of infant hemodynamic activity report the oxyHb responses because these data have higher signal-to-noise ratio than either deoxyHb or totalHb responses, and studies that do report deoxyHb activity have found inconsistent trends (Lloyd-Fox et al., 2010). However, a more complete understanding of infant metabolic activity requires that both oxyHb and deoxyHb activity be reported. In this study, the deoxyHb activation is reasonably consistent with the oxyHb activation. The fact that fewer channels show significant correlations with temperament could be due to the smaller amplitude of the deoxyHb response; there is less variation in the maximum amplitude of deoxyHb activity and thus fewer meaningful correlations.

Overall, these results provide additional evidence that infant temperament, as measured by the Infant Behavior Questionnaire, is associated with individual differences in the neural response to emotional faces. A previous study showed that NE correlated positively with a greater Nc to happy faces (Martinos et al., 2012), whereas our results demonstrate that lower NE scores are associated with greater activation to the happy face stimuli. Because the present study did not assess infants’ allocation of attention to the face stimuli, these results cannot be directly compared to previous studies (de Haan et al., 2004; Martinos et al., 2012). However, the accumulating evidence suggests that the association between infant temperament and individual differences in emotional face processing is a fruitful topic for further investigation.

### Frontal Asymmetry in fNIRS Response to Happy Face Stimuli

In analyzing the effects of hemisphere and temperament on oxyHb activity in these data, we found that there was a frontal asymmetry effect detectable with fNIRS. There was no main effect of hemisphere on oxyHb activation, but there was an interaction with NE group in the expected direction. The low-NE infants preferentially activated the left hemisphere, which is consistent with previous findings that the left hemisphere is preferentially activated during approach behaviors (Davidson, 1993), but there was no difference in hemispheric activation for high-NE infants. In response to happy faces, low-NE infants seem to demonstrate an asymmetrical approach response, but this lateralization effect is absent in high-NE infants.

The asymmetry effect measured in the left and right OFC is likely to be a response elicited by the happy face stimuli (state-dependent) that is stronger in infants with low-NE temperament than those with high-NE temperament (trait-dependent). An investigation of regional specificity in EEG asymmetry found that the effects in frontopolar recordings were more transient and were assumed to reflect OFC activity. In contrast, asymmetries calculated for dorsolateral, temporal, and parietal areas were more stable over time (Papousek and Schulter, 1998). Previous research confirms that emotional face stimuli elicit state-dependent frontal EEG asymmetry, even in infants (Davidson and Fox, 1982; Ekman et al., 1990). It is thought that asymmetrical cortical activation might be caused by differential inputs to the two hemispheres of the cortex from subcortical structures, particularly the amygdala (Kagan and Snidman, 2004). The OFC is strongly innervated by the amygdala, and it is reasonable that it would receive transient state-dependent inputs to each hemisphere.

What neurobiological activity might be driving the asymmetrical left-hemisphere activation in low-NE infants? Traditionally, frontal asymmetry is measured using EEG. In this measure, increased cortical activation is associated with desynchronization of the neural activity that produces the alpha wave, resulting in reduced power in the alpha frequency band (Davidson, 2004; Kagan and Snidman, 2004). Studies that simultaneously record electrical activity (using EEG) and metabolic activity (using fMRI or PET) have shown that alpha power is inversely correlated with high metabolism in several cortical brain regions, providing justification for using alpha power and hemodynamic activity as alternative measures of brain activation (Oakes et al., 2004). In the present study, the increased oxyHb concentrations in the left prefrontal area, relative to the right, suggest that left-hemispheric neural activation is relatively greater. As the neurons fire at a greater rate and consume greater amounts of oxygen, greater amounts of oxyHb are delivered to the local cortical area, and are detected by the fNIRS recording.

This finding provides insight into the development of hemispheric asymmetry in emotional face processing. Previous work has shown that adults’ responses to emotional faces are lateralized (Davidson, 1993; Fusar-Poli et al., 2009), and the present experiment provides evidence that this laterality is present as early as 7 months of age, and that it can be measured with fNIRS. Because of the spatial resolution of fNIRS, this technique could prove useful in parsing out the functional divisions of the PFC, and the development of these functions over the first years of life. Furthermore, studying the typical development of hemispheric asymmetry in emotional face processing will reveal information about the atypical development of these processes (Davidson, 1993).

### Limitations and Future Directions

In the current study, we used a parent report measure of infant temperament. Gartstein and Rothbart (2003) have discussed at length the limitations of various temperament assessments: parent report measures are less controlled than laboratory observation, but on the other hand, the novel setting of the laboratory could influence infants’ behavior (Putnam et al., 2008). Future studies should employ both parent-report survey data and laboratory observational assessment of temperament. Furthermore, we examined only the hemodynamic responses to happy face stimuli. Future analyses of the neural responses to neutral expressions and to other emotional expressions would provide additional insight into the interaction between temperament and prefrontal brain activity. Finally, our analysis included only one age of participant. The investigation of whether, when, and how individual temperaments change over time, from infancy into adulthood, is an important direction of future study, especially as it informs our understanding of how anxiety, depression, and social pathologies develop. Measuring the prefrontal responses to emotional faces in infants of other ages would provide information about how neural activity develops over the first year of life and would provide useful context for the interpretation of our results.

## Author Contributions

CN and MR developed the concept and, with AW, designed the experiments. AW, RV, and MR collected data, with assistance from other research assistants in the lab. KP, AW, RV, and MR analyzed the data. MR prepared the manuscript, and all authors contributed to critical revisions of the paper.

## Conflict of Interest Statement

The authors declare that the research was conducted in the absence of any commercial or financial relationships that could be construed as a potential conflict of interest.

## Abbreviations

deoxyHb: deoxyhemoglobin
EEG: electroencephalography
ERP: event-related potential
FFA: fusiform face area
fMRI: functional magnetic resonance imaging
fNIRS: functional near-infrared spectroscopy
lPFC: lateral prefrontal cortex
mPFC: medial prefrontal cortex
NE: negative emotionality
OFA: occipital face area
OFC: orbitofrontal cortex
oxyHb: oxyhemoglobin
PFC: prefrontal cortex
R-IBQsf: Revised Infant Behavior Questionnaire Short Form
RM-ANOVA: repeated measures analysis of variance
S/E: Surgency/Extraversion
STS: superior temporal sulcus
totalHb: total hemoglobin
